# A low-molecular-weight compound exerts anticancer activity against breast and lung cancers by disrupting EGFR/Eps8 complex formation

**DOI:** 10.1186/s13046-019-1207-y

**Published:** 2019-05-22

**Authors:** Meifang Li, Jilong Yang, Lenghe Zhang, Sanfang Tu, Xuan Zhou, Ze Tan, Weijun Zhou, Yanjie He, Yuhua Li

**Affiliations:** 1 Department of Hematology, Zhujiang Hospital, Southern Medical University, No. 253 GongyeDadaoZhong, Guangzhou, Guangdong 510282 People’s Republic of China; 2 State Key Laboratory of Chemo/Biosensing and Chemometrics, College of Chemistry and Chemical Engineering, Hunan University, Changsha, 410082 People’s Republic of China

**Keywords:** Epidermal growth factor receptor (EGFR), Epidermal growth factor receptor pathway substrate 8 (Eps8), Breast cancer, NSCLC, small-molecule inhibitor

## Abstract

**Background:**

Epidermal growth factor receptor (EGFR) and epidermal growth factor receptor pathway substrate 8 (Eps8) have been widely reported to be expressed in various tumors. Eps8 is an important active kinase substrate of EGFR that directly binds to the juxtamembrane (JXM) domain of EGFR to form an EGFR/Eps8 complex. The EGFR/Eps8 complex is involved in regulating cancer progression and might be an ideal target for antitumor therapy. This study focused on the screening of small-molecule inhibitors that target the EGFR/Eps8 complex in breast cancer and non-small cell lung cancer (NSCLC).

**Methods:**

In silico virtual screening was used to identify small-molecule EGFR/Eps8 complex inhibitors. These compounds were screened for the inhibition of A549 and BT549 cell viability. The direct interaction between EGFR and Eps8 was measured using coimmunoprecipitation (CoIP) and JXM domain replacement assays. The antitumor effects of the inhibitors were analyzed in cancer cells and xenograft models. An acute toxicity study of EE02 was performed in a mouse model. In addition, the effect of the EE02 inhibitor on the protein expression of elements downstream of the EGFR/Eps8 complex was determined by western blotting and protein chip assays.

**Results:**

In this study of nearly 390,000 compounds screened by virtual database screening, the top 29 compounds were identified as candidate small-molecule EGFR/Eps8 complex inhibitors and evaluated by using cell-based assays. The compound EE02 was identified as the best match to our selection criteria. Further investigation demonstrated that EE02 directly bound to the JXM domain of EGFR and disrupted EGFR/Eps8 complex formation. EE02 selectively suppressed growth and induced apoptosis in EGFR-positive and Eps8-positive breast cancer and NSCLC cells. More importantly, the PI3K/Akt/mTOR and MAPK/Erk pathways downstream of the EGFR/Eps8 complex were suppressed by EE02. In addition, the suppressive effect of EE02 on tumor growth in vivo was comparable to that of erlotinib at the same dose.

**Conclusions:**

We identified EE02 as an EGFR/Eps8 complex inhibitor that demonstrated promising antitumor effects in breast cancer and NSCLC. Our data suggest that the EGFR/Eps8 complex offers a novel cancer drug target.

## Background

Cancer is hallmarked by the accumulation of genetic variations and the loss of normal cellular regulatory processes and is a main cause of death worldwide [1, 2]. Although chemotherapy is the cornerstone of systemic cancer therapy, it has a modest effect on overall survival [3]. Lung cancer is still the leading cause of cancer-related death worldwide, and breast cancer is the most common type of noncutaneous malignancy and the leading cause of cancer-related death in women worldwide [4–6]. Thus, there is a great need to develop novel therapeutic modalities to improve survival rates [7].

Epidermal growth factor receptor (EGFR), also known as ErbB1 or HER1, is a member of the receptor tyrosine kinase (RTK) family of cell surface receptors [8]. EGFR regulates differentiation, apoptosis, cell cycle progression, development, and transcription [9–11]. EGFR hyperactivity, caused either by mutation or overexpression of the ligand or receptor, contributes to a variety of human cancers [12]. EGFR consists of an extracellular domain, a single hydrophobic transmembrane segment, an intracellular portion with a juxtamembrane (JXM) segment, a protein kinase domain, and a carboxyterminal tail [8].

Recent studies have shown that the presence of the JXM domain is necessary for the full catalytic activity of EGFR [13–15]. A study by Thiel and Carpenter showed that compared with the activity of constructs containing the full-length intracellular domain (ICD) containing the JXM domain, the activity of intracellular EGFR constructs lacking the JXM domain was reduced by approximately 95% [15]. In a later study in the Carpenter laboratory, scanning alanine mutagenesis experiments showed that ICDs constructed with mutations at 36 different residues in the JXM domain reduced EGFR activity by approximately 50% [15]. Two of the mutations (V665A and L680A) were able to significantly reduce the activity of full-length EGFR [15]. These results show that most of the amino acids (aa) within the JXM domain play important roles in supporting the activation of the kinase domain [13–17]. The JXM domain not only links the transmembrane region to the kinase domain but also contains the initial 13 amino acids involved in the EGFR dimerization process [18]. Studies have shown that the JXM domain plays an activating role in EGFR, and the deletion of half of the C-terminal amino acids (664–682) in the JXM domain will affect the formation of EGFR dimers, resulting in the loss of EGFR kinase activity [13]. JXM domain V665 M or L679F amino acid mutations can increase EGFR activity due to the formation of a more stable EGFR dimer [13]. According to Sinclair et al.*,* the peptide E1 derived from the JXM domain can bind to the EGFR JXM domain and effectively reduce EGFR dimerization, which thereby affects EGFR activity and reduces cell viability [19]. Boran once demonstrated that the JXM domain of EGFR is essential to the activation of EGFR, and the JXM domain activates and regulates EGFR activation and is a potential target for the development of new EGFR inhibitors [14].

Epidermal growth factor receptor pathway substrate 8 (Eps8) is an important active kinase substrate of EGFR [2, 20]. EPS8 is efficiently phosphorylated by various tyrosine kinases, both receptor (RTK) and nonreceptor types, and is a typical signaling protein, with a molecular weight of 97 kDa and containing a phosphotyrosine binding protein (PTB) domain, an Src homology 3 (SH3) domain and a sterile alpha-pointed (SAM-PNT) domain [21, 22]. Eps8 is frequently overexpressed in breast, lung and other malignancies but rarely in normal tissues [23–26]. Further studies of EPS8 have revealed that a domain that encompasses amino acids 298 to 362provides a binding surface for the JXM domain of EGFR [27]. Studies by Fazioli et al. and Castagnino et al. have shown that Eps8 directly binds to the JXM domain of EGFR and is phosphorylated, which activates a series of downstream signaling pathways [20, 27], and thus promotes tumor progression. Furthermore, the aberrant expression of Eps8 often suggests anunfavorable prognosis for cancer patients [20, 27, 28]. Therefore, Eps8 is considered a novel potential target for specific cancer therapy.

Eps8 directly binds to the JXM domain of EGFR and forms an EGFR/Eps8 complex. Studies on the EGFR/Eps8 complex in malignancies are limited. In the present study, we focused on the EGFR/Eps8 complex as a promising tumor target for cancer therapy. The Eps8-derived 9-amino acid peptide 327, which partly mimics the EGFR binding region of Eps8, functions as a protein-protein interaction module that can disrupt the EGFR/Eps8 complex, prevent the activity of the downstream EGFR pathway, and exert antitumor effects [2]. In our opinion, there is another approach to disrupt the EGFR/Eps8 complex. Direct inhibition of the EGFR/Eps8 complex by using drug-like, nonpeptide small molecules have several advantages, including blocking the activity mediated by EGFR/Eps8 complex activation, improved cell permeability and better in vivo stability and bioavailability. Based on the high-resolution X-ray 3D crystal structure of JXM and the kinase domain of EGFR, the JXM domain is critical for EGFR activation and acts as a binding site for Eps8 [13, 27]. Therefore, we hypothesize that a small molecule that binds to the JXM domain of EGFR may directly disrupt EGFR/Eps8 complex formation and block the downstream pathway of the complex. In this work, we report the discovery of an EGFR/Eps8 complex small-molecule inhibitor through virtual database screening.

## Methods

### Structure-based virtual screening

To identify potential candidate compounds that can disrupt EGFR/Eps8 complex formation, the crystal structure of EGFR solved at 2.8-Å resolution was retrieved from the Protein Data Bank (PDB ID code 3GOP) (DOI: 10.2210/pdb3GOP/pdb) [29] and used in this study. The chemical databases used in our virtual screening were provided by TopScience Co., Ltd. Collectively, these databases offer a collection of 390,000 small-molecule organic compounds. The chemical catalogs provide only 2D chemical structures; the 3D structural models were generated by using SYBYL software (Version 7.3, Tripos Associates, St. Louis) with the standard settings. The molecular docking program SYBYL was used to perform the virtual screening. The binding cavity in the EGFR JXM domain was the region targeted for docking. SYBYL software was used to assign the standard AMBER (refers to a set of molecular mechanical force fields for the simulation of biomolecules) atomic partial charges on the EGFR protein and the Gasteiger-Hückel atomic partial charges on each ligand molecule to be docked. The parameters used for modeling docking, which controlled how the docking was performed in our work, were standard settings according to the guidelines of the software [30]. Each molecule in the assessed databases with a molecular weight between 200 and 1000 was docked into the targeted binding site of EGFR. The top 5% scored compounds from each database, as selected by SYBYL, were extracted and combined to provide a total of 18,000 candidate compounds. The preselected 18,000 compounds then were reranked according to their binding affinities as estimated by the total score. Of the best-scored 50 compounds selected by the total score from the 18,000 compounds, physical samples of 29 of these compounds were purchased from TopScience Co., Ltd. PyMOL software (Version 1.6.X, Open-Source) was obtained from the PyMOL website (https://pymol.org) [31].

### Cell lines and culture conditions

The non-small cell lung cancer cell lines (A549、H460、H1975), breast cancer cell lines (MCF-7、BT549、MDA-MB-468) and multiple myeloma cell line (IM-9) were kept in our laboratory (Hematological Laboratory of Zhujiang Hospital, Guangzhou, China) and genotyped to verify their authenticity. Human normal lung epithelial cell line (BEAS-2B) and human normal mammary epithelial cell line (MCF-10A) were purchased from FuHeng Cell Center (Shanghai, china) and genotyped to verify their authenticity. Normal PBMCs were obtained from 5 unrelated healthy donors at Southern Medical University (Guangzhou, China). MCF-10A cell line was cultivated in MEpiCM medium (Cat No. 7611, ScienCell, San Diego, California, USA) and other cell lineswere cultivated in DMEM or RPMI 1640 medium (Invitrogen, Grand Island, NY, USA) with 10% fetal bovine serum (FBS; Invitrogen) at 37 °C with 5% CO_2_.

### Compounds and growth-inhibiton assay

For testing different compounds, A549 and BT549 cells were plated in a 96-well-plate (5 × 10^3^ cells/well) and incubated for 24 h before treatment. Then cells were exposed to the compounds for 24 h at a final concentration of 10 μM. After treatment, 10 μl of CCK-8 reagent (Dojindo Laboratories, Japan) was added to each well, and cells were incubated for 3 h at 37°C and 5% CO_2_. The optical density (OD) was analyzed at 450 nm. The data obtained are presented as percentage viability in the graph.

### Plasmid constructs and the creation of stable EGFR-overexpressing cell lines

The wild-type EGFR (wt-EGFR) sequence was obtained through an NCBI search, the whole length of the gene was synthesized by GENEWIZ, Inc. (Germantown, MD), and the two ends were cloned into the vector PCD531B (SBI) using the restriction site NheI//NotI. At the same time, 30 amino acids in the JXM domain (aa: 650–679) were replaced with a 10 GGS repeating sequence to obtain the new gene sequence mutant-type EGFR (mut-EGFR), which was produced by GENEWIZ, Inc. (Germantown, MD), and both ends were cloned into the vector PCD513B (SBI) by using the restriction site NheI//NotI [32]. The sequence integrity of the completed two vectors were ensured by sequencing. MCF-7 cells were transfected with wt-EGFR, mut-EGFR or PCD531B empty vector using Lipofectamine 2000 (Invitrogen) following the manufacturer’s protocol. Twenty-four hours after transfection, the culture medium was replaced by medium supplemented with 1.0 μg/ml puromycin. Two or 3 days later, the cells that grew to approximately 90% confluency were passed and seeded at a high dilution ratio into 150-mm cell culture dishes. The medium containing puromycin was replaced every 2–3 days until colonies of cells appeared approximately 2 weeks after the initial seeding. Several colonies in each transfection group were selected for further experiments and designated as MCF-7-NC, MCF-7-wt-EGFR or MCF-7-mut-EGFR.

### Creation and characterization of stable EGFR knockdown cell lines

EGFR expression was stably knocked down in A549, H460 and MDA-MB-468 cells via RNA interference (RNAi). The annealed oligonucleotide fragments encoding short hairpin transcripts corresponding to EGFR were as follows: CCGGGCAGATCATCAGAGGAAATATCTCGAGATATTTCCTCTGATGATCTGCTTTTT and CCGGGGAGATAAGTGATGGAGATGTCTCGAGACATCTCCATCACTTATCTCCTTTTT. The nontargeting empty plasmid was used as the control shRNA plasmid. According to the manufacturer’s instructions, A549, H460 and MDA-MB-468 cells (2 × 10^5^cells/well in six-well plates) were transfected separately with the control shRNA plasmid or the EGFR shRNA plasmid using Lipofectamine 2000 (Invitrogen). After 24 h, the culture medium was replaced with medium supplemented with puromycin at a selection concentration. Several colonies in each transfection group were selected for further experiments and designated as A549-NC, A549-EGFR/shRNA1, A549-EGFR/shRNA2, H460-NC, H460-EGFR/shRNA1, H460-EGFR/shRNA2, MDA-MB-468-NC, MDA-MB-468-EGFR/shRNA1 or MDA-MB-468-EGFR/shRNA2.

### Cell proliferation/survival assays and apoptosis measurements

To test the effects of EE02 on the biological activities of tumor cells and normal cells, tumor cell lines, normal cell lines and normal PBMCs were plated in a 96-well plate (5 × 10^3^cells/well) and incubated for 24 h before treatment. All cells were then incubated with different concentrations of EE02 (0, 1, 2.5, 5.0, 7.5 or 10 μM) for 24 h.CCK-8 (Dojindo Laboratories, Japan) assays were carried out according to the manufacturer’s instructions. Colonyformation assays were also performed. Cells (A549 and H460 were 300 cells/well, BT549 and MCF-7 were 500 cells/well) were seeded into six-well plates and treated with different concentration’s EE02 or DMSO on the following day. After 2 weeks, the cells were fixed with4% formaldehyde and washed with PBS when colonies were visible. Crystal violet was added to the plates to stain the colonies. The number of apoptotic cells was analyzed by flow cytometry (BD Bioscience) using Annexin V and propidium iodide (PI; BD Biosciences). Cells (1 × 10^6^ cells/well) were seeded in six-well plates and exposed to 10 μM EE02 or DMSO, and they were harvested and processed after 24 h according to the manufacturer’s instructions [33].

### Western blot analysis

All prepared cells were homogenized in IP lysis (CO-IP kit, Thermo Scientific, Cat No. #26149), and debris was removed by centrifugation at 12,000 g for 10 min at 4 °C. The protein concentrations were determined using a Bradford protein assay kit (Beyotime, China, Cat No. #KGP902). After addition of loading buffer, protein samples were electrophoresed, transferred to PVDF membranes (0.2 μm, Millipore, Bedford, MA), and subsequent blocked. The membranes were immunoblotted with rabbit anti-human primary antibody overnight at4 °C. Antibodies of EGFR, Erk, p-Erk, Akt, p-Akt (ser473) and GAPDH were obtained from Cell Signaling Technology (Cat No. #4267, #9102, #9101, #9272, #9271 and #5174 respectively; dilution ratio was 1:1000). Antibody targeting EPS8 was purchased from Biosciences (Cat No. #610143; dilution ratio was 1:1000). After four washes with TBST, the blots were incubated with horseradishperoxidase (HRP)-conjugated secondary antibodies at room temperature for 1 h, and the HRP signal was detected using enhanced chemiluminescence (Pierce Biotechnology, Rockford, IL, USA).

### AKT-1 phosphorylation antibody array and CoIP

We performed human AKT-1 phosphorylation antibody arrays, which included 18 different antibodies (RayBiotech, Inc., USA) [34, 35]. All the steps are carried out according to the manufacturer’s instructions. Briefly, MDA-MB-468 cells from different groups (EE02 10 μM and DMSO control) were lysed and the total proteins were purified using the Cell and Tissue Protein Extraction Reagent (Kangchen, China). The protein concentrations were tested using a Bradford protein assay kit (Beyotime, China, Cat No. #KGP902). A total of 380 μg protein extract was added into each well and incubated for 3 h at room temperature. Then, the antibody arrays membranes were washed and incubated with HRP-Anti-Rabbit IgG antibody at room temperature for 2 h, and the HRP signal was detected using ImageQuant LAS4000 Scanner (GE Healthcare Corporate, USA). For the coimmunoprecipitation (CoIP) of endogenous proteins, 10 μg of anti-EGFR (Cat No. #4267, CST) or IgG (Cat No. #2729, CST) antibody was added to AminoLink plus coupling resin (Thermo Fisher Scientific, Pierce CoIP kit, Cat No. #26149) for antibody immobilization at room temperature for 100 min. Cells were lysed in cell lysis buffer at 4 °C for 30 min [36]. A total of 1000 μg protein extract was added into each tube of control agarose resin and incubated for 1 h at 4 °C. Then, the protein was centrifuged from the control agarose tube, and the protein-containing liquid was added to the antibody incubation tube, and the mixture was incubated at room temperature for 2 h. Immunoprecipitants were separated by SDS-PAGE after washing with the same buffer and were analyzed by immunoblotting with an anti-Eps8 antibody [37].

### Chemicals

Erlotinib was purchased from Selleckchem, dissolved in DMSO at a final concentration of 50 mg/ml and stored at − 20 °C.

### Acute intraperitoneal toxicity

Thirty-five male and thirty-five female specific pathogen-free (SPF) BALB/c mice were randomly assigned to seven groups: six treatment groups and one vehicle control group, with five male and five female mice per group. The vehicle control group received DMSO in a volume of 5 mL/kg body weight (b.w.) by intraperitoneal injection. EE02 was dissolved in DMSO and administered to the mice at doses of 75.00, 86.25, 99.19, 114.19, 131.75 or 151.51 mg/kg b.w., and all animals were observed every day for symptoms and mortality for two weeks. The vehicle control group was observed at the same time. All of the surviving animals were euthanized at the end of the study. The vital organs of both the mouse that died and those that were euthanized were individually observed for overt pathology by necropsy, and the 50% lethal dose (LD_50_) was calculated by the Bliss method on day 14. The vital organs were removed, fixed, and embedded in paraffin for histopathological analyses [38, 39].

### Xenograft tumor model

All animal experiments complied with Southern Medical University’s Policy on the Care and Use of Laboratory Animals. Five-week-old athymic BALB/c nu/nu male mice were used for in vivo experiments. The animals were housed at a constant room temperature with a 12 h light/12 h dark cycle and fed a standard rodent diet and water. H460 cells were harvested and injected subcutaneously (5 × 10^6^ cells in 100 μl of phosphate-buffered saline (PBS)) into the mice. Treatment began after the size of the H460 tumors reached 50 mm^3^, and the treatments consisted of intraperitoneal (i.p.) injections of DMSO, EE02 (5 mg/kg b.w. in 100 μl of DMSO), EE02 (10 mg/kg b.w. in 100 μl of DMSO) or erlotinib (10 mg/kg b.w. in 100 μl of DMSO). The treatment was performed every 2 days. Tumor growth was monitored by a digital caliper, and the maximum tumor volume was not allowed to exceed 1500 mm^3^. At the end of the experiment, the animals were sacrificed, and the tumors were removed. The tumor volumes were determined by measuring the tumor length (L) and width (W) and calculating the volume (V = 0.5 × L × W^2^), [40]. Tumor samples were excised, fixed, and embedded in paraffin for immunohistochemical (IHC) analyses [41, 42].

### Statistical analysis

Statistical significance was evaluated using SPSS 21.0 software. *P* < 0.05 was considered statistically significant. * Represents P < 0.05, ** represents *P* < 0.01, and *** represents *P* < 0.001.

## Results

### Discovery of EE02 through structure-based virtual database screening

**Fig. 1:**
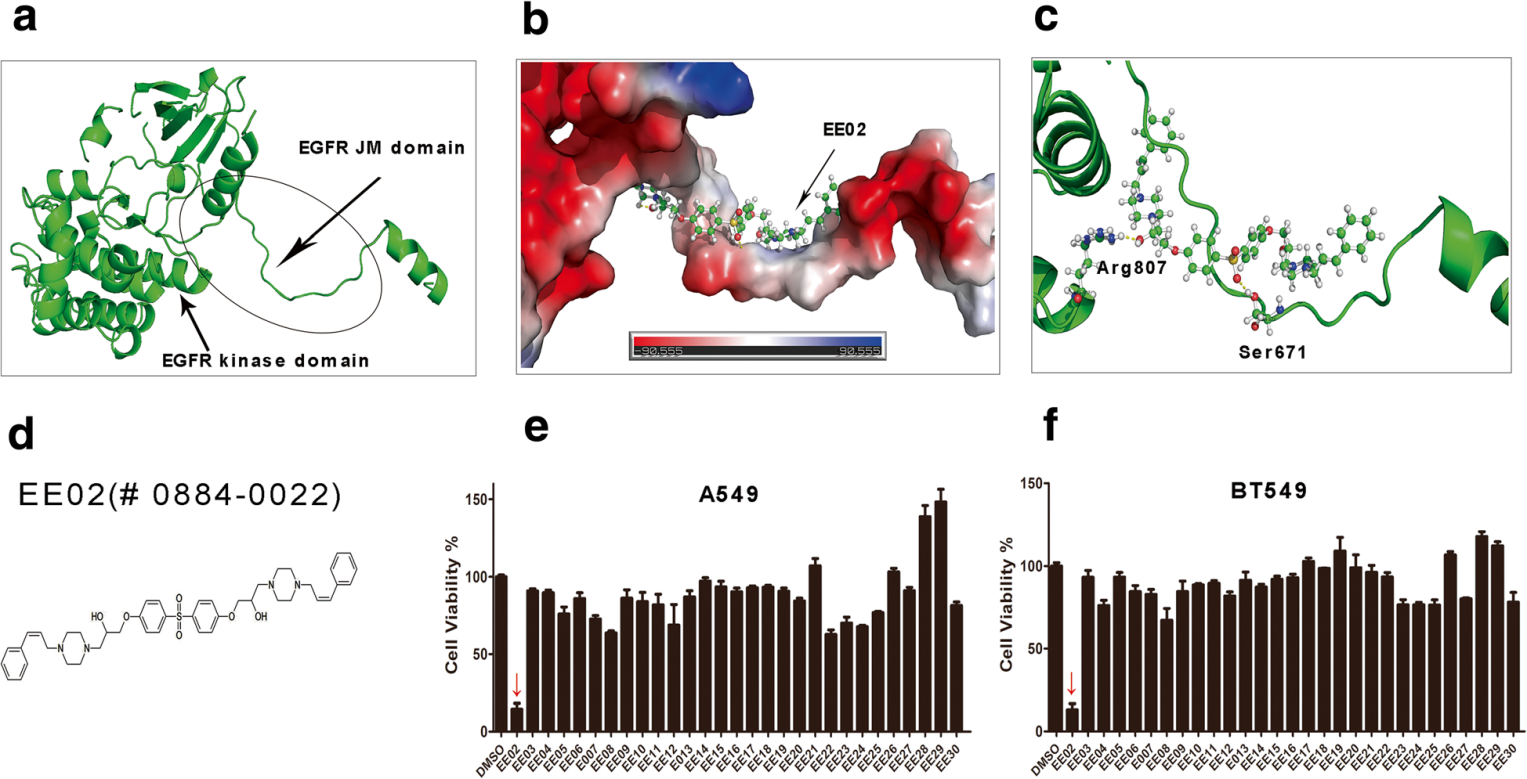
Schematic diagrams of the modeling used for the structure-based virtual database screening. **a** JXM and kinase domains of the EGFR protein. The structure is based on the Protein Data Bank entry 3GOP. The circled region indicates the target JXM binding site used in our virtual screening study. **b** The Predicted model of EE02 binding to the EGFR JXM and kinase domains. EE02 is rendered as a ball-and-stick model. The molecular surface of the EGFR JXM and kinase domains is colored to indicate electrostatic potentials: red indicates the most positively charged regions, and blue indicates the most negatively charged regions. **c** Specific hydrogen bonds formed between the EGFR JXM and kinase domains and EE02. The binding model was predicted by SYBYL. Only the residues that form hydrogen bonds with EE02 are shown in the explicit atomic models. **d** EE02 structure. **e**, **f** A549 and BT549 cells were used for the initial EGFR/EPS8 complex small-molecule inhibitor screening. The cells were treated with 10 μM EE02 as well as other small-molecule compounds for 24 h, and then the cells were harvested for analysis by a CCK-8 assay (*n* = 3). **a-c** were generated by using PyMOL

The EGFR binding region of EPS8 is predicted to target the JXM domain of EGFR. We hypothesized that a small molecule that binds to the JXM domain of EGFR will compete with EPS8, consequently blocking the EGFR/EPS8 interaction. With the aid of structure-based virtual screening, we chose a crystal structure of EGFR (PDB ID code 3GOP) that includes the kinase region and the JXM domain. Using SYBYL software to predict and generate the docking pocket of EGFR (Fig. 1a), we narrowed our interest from a total of 390,000 compounds to 50 top candidate compounds and were able to obtain chemical samples of 29 of the top 50 compounds. We first tested the effects of these 29 molecules on the proliferation of A549 and BT549 cell lines by using CCK-8 assays in vitro. Of the 29 compounds tested, the most promising compound was EE02 (ChemDiv No. 0884–0022), which was obtained from TopScience Co., Ltd. (Fig. 1e and f). EE02 is a synthetic compound with a molecular weight of 767 (Fig. 1d). The model of EE02 binding to EGFR was generated by SYBYL software and refined by structural organization using PyMOL software. The refined model, shown in Fig. 1b and c, predicted that EE02 binds the JXM domain and the meshed area where the JXM domain intersects with the kinase domain and forms a number of hydrogen bonds with nearby residues, including Ser-671 and Arg-807 (Fig. 1c).

### EE02 significantly inhibited the viability and proliferation of EGFR-positive and Eps8-positive breast cancer and NSCLC cells

**Fig. 2:**
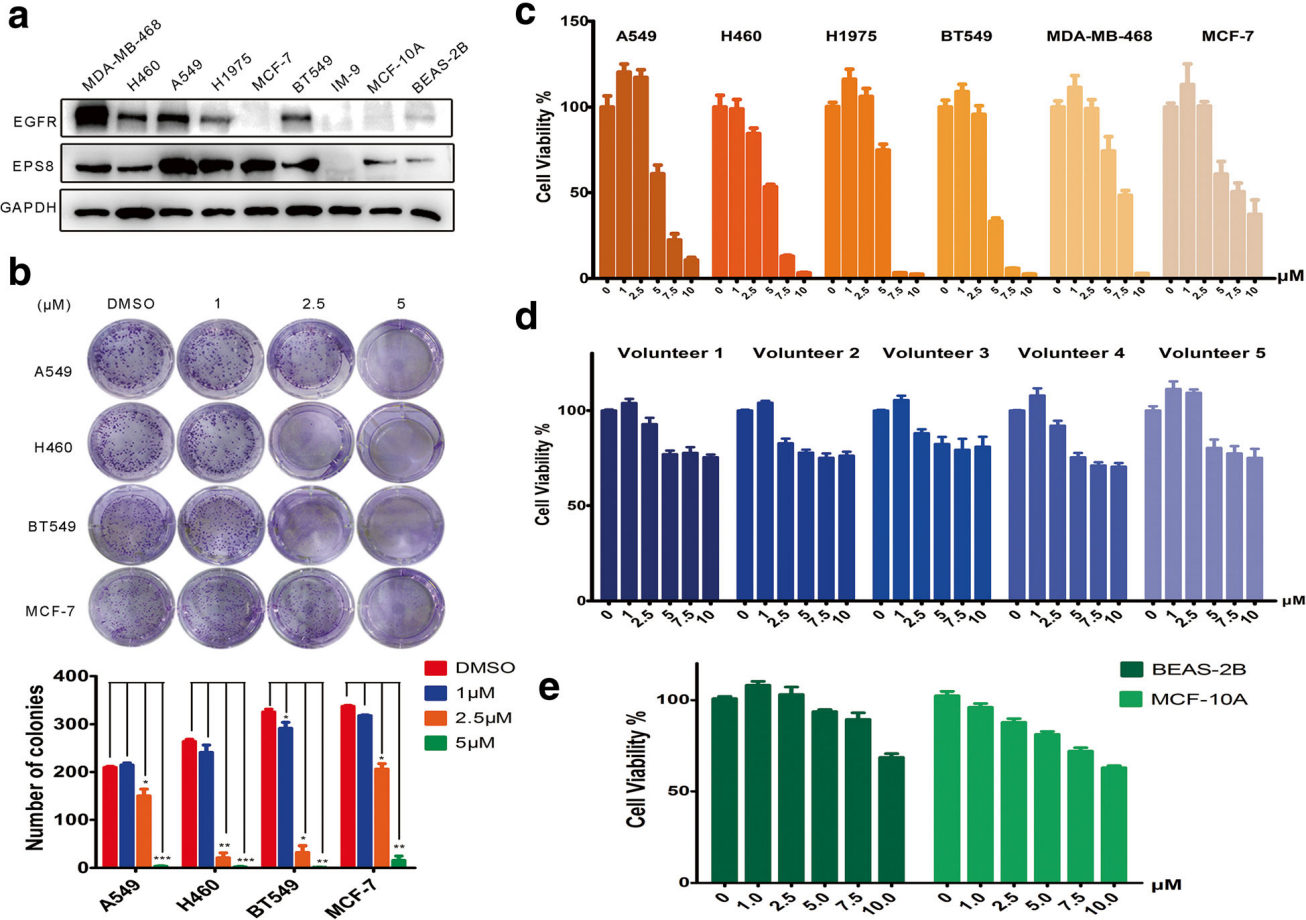
Biological activity of EE02 in vitro. **a** The expression levels of EGFR and EPS8 in different cell lines are shown. **b** A549, H460, BT549 and MCF-7 cells were seeded and treated with increasing concentrations of EE02 for 2 weeks. Colonies were counted, and images were acquired. The quantification of the colony numbers is shown. **c** Cancer cell lines were treated with EE02 for 24 h at increasing concentrations (0, 1, 2.5, 5, 7.5 and 10 μM), and then cell viability was assessed using CCK-8 assays (n = 3). **d** PBMCs from 5 untreated healthy donors were treated with increasing concentrations (0, 1, 2.5, 5, 7.5 and 10 μM) of EE02 for 24 h, and cell viability was analyzed via CCK-8 assays (n = 3). **e** Normal cell lines were treated with EE02 for 24 h at increasing concentrations (0, 1, 2.5, 5, 7.5 and 10 μM), and then cell viability was assessed using CCK-8 assays (n = 3)

Initially, to screen the expression levels of EGFR and EPS8 in cancer and normal cell lines, we performed a western blot analysis. The results indicated EGFR overexpression in the MDA-MB-468, H460, A549 and BT549 cell lines; moderate EGFR expression in the H1975 cell line; and very low EGFR expression in the MCF-7, IM-9, MCF-10A and BEAS-2B cell lines. EPS8 was overexpressed in all six breast cancer and NSCLC cell lines (MDA-MB-468, H460, A549, H1975, MCF-7 and BT549), moderate expressed in normal cell lines (MCF-10A and BEAS-2B) and very low expressed in IM-9 cell line (Fig. 2a). Then, to assess the activity of EE02, A549, H460, H1975, BT549, MDA-MB-468 and MCF-7 cells were treated for 24 h with increasing concentrations of the inhibitor EE02 (0–10 μM). The dose-dependent antiproliferative effect of EE02 was determined using CCK-8 assays (Fig. 2c). However, at a 10 μM concentration, the antiproliferative effect of the inhibitor EE02 on the proliferation of the A549, H460, H1975, BT549 and MDA-MB-468 cancer cells was significantly stronger than that on the proliferation of the MCF-7 cells, which overexpress EPS8 but not EGFR (*P* = 0.003, 0.008, 0.008, 0.008 and 0.009, respectively, t-tests) (Fig. 2a and c). We then examined the effect of EE02 on the colony formation capability of the breast carcinoma and NSCLC cells and found that the inhibitor EE02 significantly reduced colony numbers of A549 (*P* < 0.001, one-way ANOVA), H460 (*P* < 0.001, one-way ANOVA), H1975 (*P* < 0.001, one-way ANOVA) and BT549 (*P* < 0.001, one-way ANOVA) in a dose-dependent manner (Fig. 2b). At 5 μM, the inhibitory effect of EE02 on the colony formation capability of the A549, H460 and BT549 cancer cells was stronger than that on the colony formation capability of the MCF-7 cells (Fig. 2b). Moreover, EE02 did not result in significant suppression in normal cell lines (MCF-10A and BEAS-2B, less than 40% suppression) and peripheral blood mononuclear cells (PBMCs) from 5 untreated healthy volunteers (less than 30% suppression), suggesting that EE02 possesses specificity toward cancer cells that overexpress EGFR and EPS8 (Fig. 2d and e).

### EE02 promotes apoptotic cell death

**Fig. 3:**
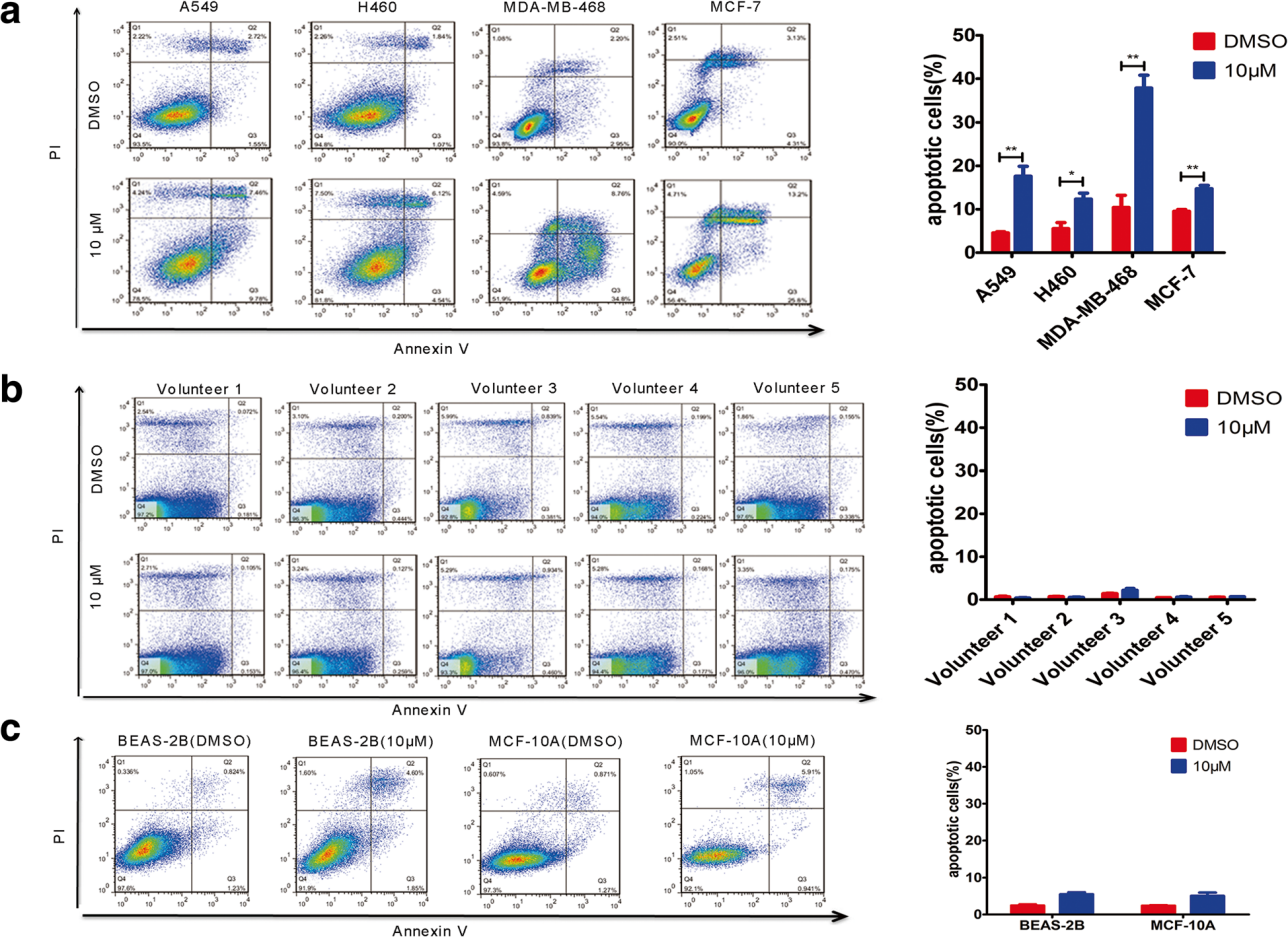
Effect of EE02 on apoptosis of cells in vitro. **a** Cancer cells were incubated with DMSO or EE02 (10 μM) for 24 h and then analyzed for apoptosis using annexin V/PI staining assays. The quantification of the annexin V/PI staining is shown. **b** PBMCs from 5 untreated healthy donors were incubated with DMSO or EE02 (10 μM) for 24 h and then analyzed for apoptosis using annexin V/PI staining assays. The quantification of the annexin V/PI staining is shown. **c** Normal cell lines were incubated with DMSO or EE02 (10 μM) for 24 h and then analyzed for apoptosis using annexin V/PI staining assays. The quantification of the annexin V/PI staining is shown

To examine the apoptotic effect of EE02, A549, H460, MDA-MB-468 and MCF-7 cells were treated with DMSO or EE02 for 24 h and then stained with annexin V and propidium iodide (PI). As is shown in Fig. 3a, after 24 h of exposure to 10 μM EE02, a significant increase in the apoptosis rates of A549 (*P* = 0.005, t-test), H460 (*P* = 0.026, t-test), MDA-MB-468 (*P* = 0.003, t-test) and MCF-7 (*P* = 0.004, t-test) cell lines were observed (Fig. 3a). The ability of EE02 to promote apoptosis was significantly stronger in the A549, H460 and MDA-MB-468 cancer cells than in the MCF-7 cells (*P* = 0.037, 0.045 and 0.016, respectively, t-test) (Fig. 3a). Moreover, EE02 did not result in a significant promotion of apoptosis in normal cell lines (MCF-10A and BEAS-2B, less than 7% apoptotic cells) and PBMCs from 5 untreated healthy volunteers (less than 5% apoptotic cells), suggesting that EE02 possesses specificity toward cancer cells that overexpress EGFR and EPS8 (Fig. 3b and c).

### EGFR knockdown reduces the sensitivity of cancer cells to EE02

**Fig. 4:**
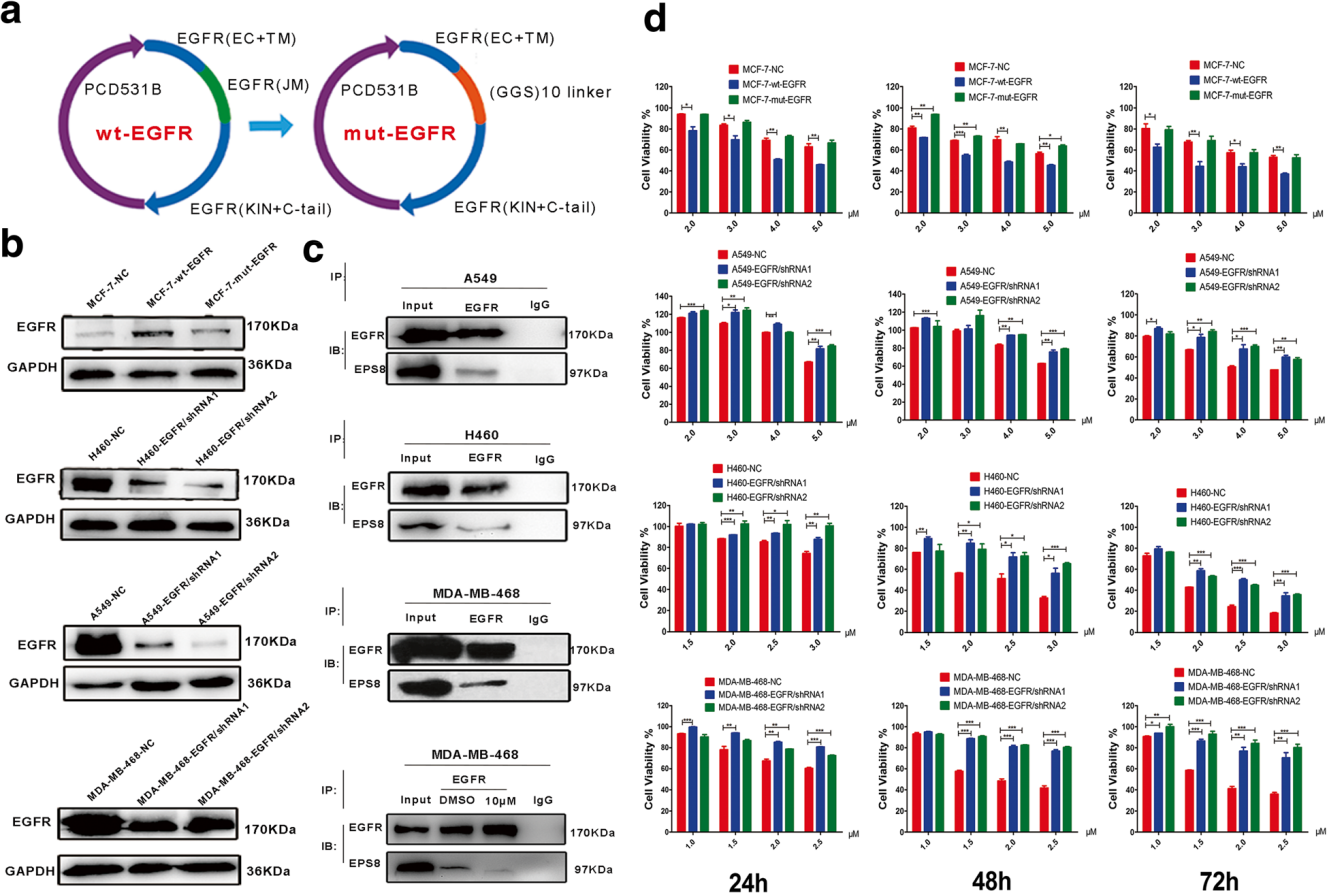
EE02 blocks the EGFR/EPS8 interaction. **a** The schematics of the plasmids used in the study encoding wild-type EGFR and mutant-type EGFR are shown. To create the mut-EGFR plasmid, the oligonucleotides encoding the EGFR JXM domain (amino acids 650–679) were replaced by an unstructured sequence encoding ten GGS repeats as described in the Methods section. **b** Cancer cell lines transfected with wt-WGFR, mut-EGFR, EGFR/shRNA1, EGFR/shRNA2 and NC shRNA were subjected to western bolt analysis for EGFR and GAPDH expression. **c** Cells were untreated or treated with EE02 or DMSO for 24 h, then the cell lysates were immunoprecipitated with an anti-EGFR antibody or an anti-IgG antibody and were immunoblotted with the indicated antibody. **d** Cancer cell lines transfected with wt-WGFR, mut-EGFR, EGFR/shRNA1, EGFR/shRNA2 and NC shRNA were treated with EE02 for 24 h, 48 h or 72 h at increasing concentrations, and then cell viability was assessed using CCK-8 assays (n = 3)

To verify that the antitumor effect of EE02 on cancer cells was related to the EGFR protein, we knocked down EGFR expression in H460, A549 and MDA-MB-468 cells; performed western blotting and gray values analysis; and compared the results with those of the vehicle-treated H460, A549 and MDA-MB-468 cells. As shown in Fig. 4b, the EGFR expression in the H460-EGFR/shRNA1 and H460-EGFR/shRNA2 cells was significantly downregulated compared to that in the H460-NC cells, and the same was true of the A549 and MDA-MB-468 cell lines. Subsequently, to detect whether the sensitivity of tumor cells to EE02 was reduced after knocking down EGFR, the cancer cells were treated for 24, 48 or 72 h with increasing concentrations of the inhibitor EE02 (H460: 0–3.0 μM; A549: 0–5.0 μM; and MDA-MB-468: 0–2.5 μM), and CCK-8 assays were then performed. The results showed that the sensitivity of the A549, H460 and MDA-MB-468 cells to the inhibitor EE02 was significantly decreased after the knockdown of EGFR expression, suggesting that the antitumor effect of EE02 is related to EGFR (Fig. 4d).

### EGFR overexpression increases the sensitivity of cancer cells to EE02

To verify that the inhibitor EE02 acts as an antitumor agent by targeting the EGFR JXM domain, we designed a mutant receptor, mut-EGFR, in which the JXM domain is replaced by a (GGS)_10_ sequence (Fig. 4a). The (GGS)_10_ sequence has been demonstrated to lack a regular secondary structure and to be very flexible. The plasmid was created as described in the Methods. Briefly, amino acids 650–679 of EGFR were replaced by the (GGS)_10_ sequence.

MCF-7 cells were transfected with wt-EGFR, mut-EGFR, or the empty vector. The transfected cells were analyzed by western blotting and gray values analysis, and the results were compared with those of the vehicle-treated MCF-7 cells. As shown in Fig. 4b, the EGFR expression levels in the MCF-7-wt-EGFR and MCF-7-mut-EGFR cells were significantly upregulated compared to those in the MCF-7-NC cells. Subsequently, to detect whether the sensitivity of tumor cells to EE02 increased after the overexpression of EGFR, the cancer cells were treated for 24 h, 48 h or 72 h with increasing concentrations of the inhibitor EE02 (0–5.0 μM), and then CCK-8 assays were performed. The results showed that the sensitivity of the MCF-7-wt-EGFR cells to the inhibitor EE02 was significantly higher than that of the other transfected cells, but there was no significant difference between the sensitivities of the MCF-7-mut-EGFR and MCF-7-NC cells. The results indicate that the antitumor effect of EE02 is mediated by targeting the EGFR JXM domain (Fig. 4d).

### EE02 blocks the EGFR/EPS8 interaction and suppresses EPS8 downstream signaling in cancer cells

Previous results have shown that EE02 inhibits the proliferation of tumor cells by targeting the JXM domain of EGFR. EGFR is a transmembrane protein, and EPS8 is an intracellular protein. We assume that Eps8 interacts with the JXM domain of EGFR, which is intracellular and close to the membrane. The coimmunoprecipitation results showed that in A549, H460 and MDA-MB-468 cells, which express both EGFR and EPS8, Eps8 interacted with EGFR to form an EGFR/EPS8 complex (Fig. 4c). Can the inhibitor EE02 disrupt the EGFR/EPS8 interaction by binding to the JXM domain of EGFR? To test this hypothesis, MDA-MB-468 cells expressing high levels of endogenous EGFR and EPS8 were treated with EE02 (10 μM) for 24 h. Coimmunoprecipitation was performed using an anti-EGFR antibody. The results indicated that the treatment with EE02 resulted in EGFR/EPS8 complex dissociation. As is shown in Fig. 4c, little EPS8 was detected in the EGFR immunoprecipitants after the treatment with the inhibitor EE02. Our results showed that EE02 is a potent inhibitor that was predicted to bind to the EGFR JXM domain and block the EGFR/EPS8 complex.

**Fig. 5:**
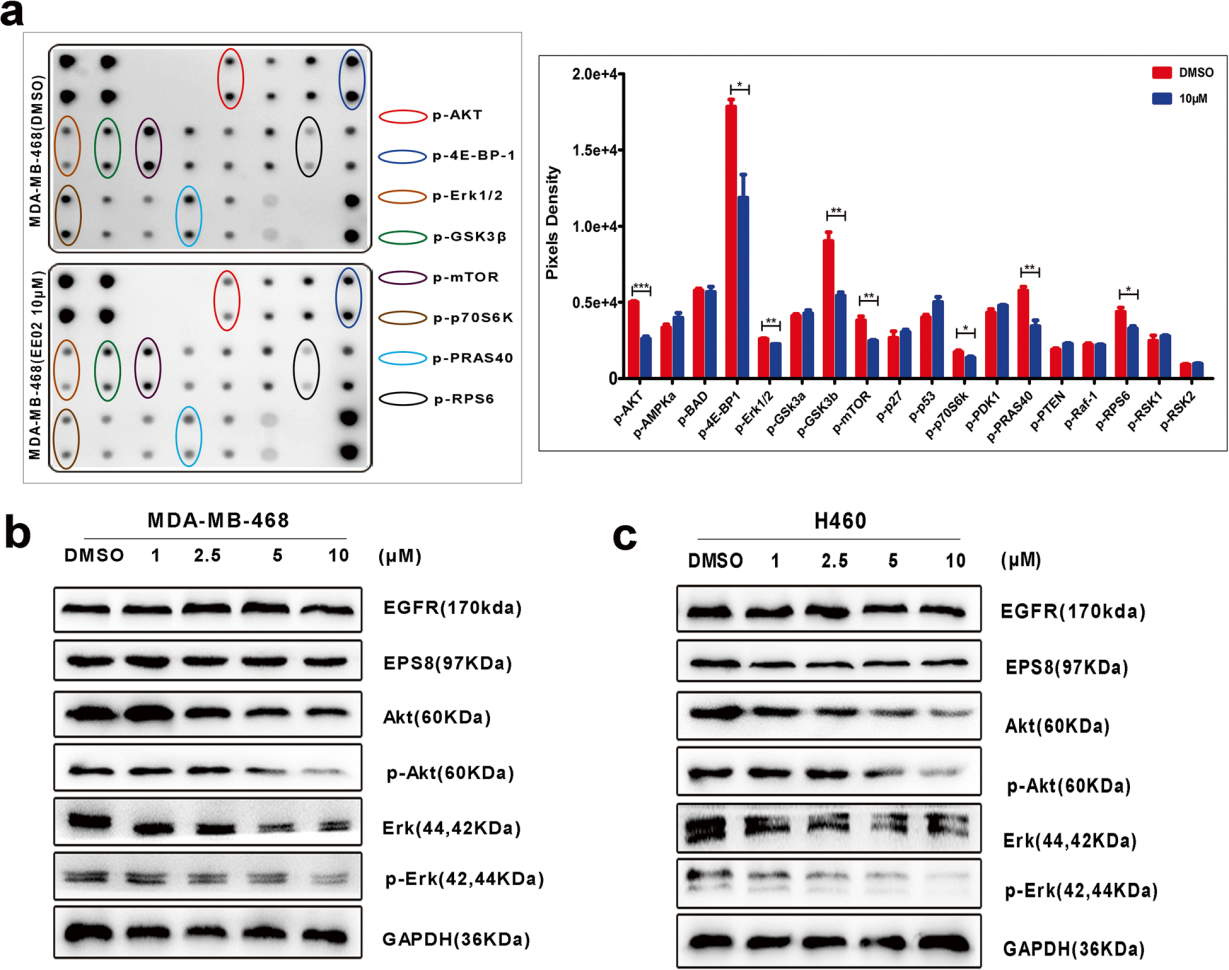
The inhibitory effects of EE02 on EGFR/Eps8 complex-associated signaling. **a** The results of protein chip assays are shown. MDA-MB-468 cells were incubated with DMSO or EE02 (10 μM) for 24 h, and then total protein was extracted for protein chip assays. **b** Western blot assays for the expression of EGFR, EPS8, Akt, p-Akt, Erk and p-Erk were performed with MDA-MB-468 cells treated with increasing concentrations of EE02 for 24 h. **c** Western blot assays for the expression of EGFR, EPS8, Akt, p-Akt, Erk and p-Erk were performed with H460 cells treated with increasing concentrations of EE02 for 24 h

EE02 blocks EGFR/EPS8 complex formation by competitively binding to the JXM domain of EGFR. What effects does EE02 have on the signaling pathway downstream of this complex? To further examine the inhibitory effect of EE02 on EGFR/EPS8 complex downstream signaling, we detected the expression of EGFR/EPS8 complex target proteins by protein chip assays. MDA-MB-468 is a breast cancer cell line that expresses high levels of EGFR and Eps8. We examined protein levels after these cells were treated with EE02 (10 μM) for 24 h. We also included a control group treated with DMSO. The protein chip results showed that the levels of p-Akt, p-4E-BP-1, p-Erk1/2, p-Gsk3β, p-mTOR, p-p70S6K, p-PRAS40 and p-RPS6 were apparently decreased after the treatment with EE02 compared with that after the treatment with DMSO, but there was no difference between the EE02 group and the DMSO control group in the expression of other proteins on these protein chips (Fig. 5a).

To further identify the inhibitory effect of EE02 on downstream MAPK/Erk and PI3K/Akt signaling, we measured EGFR, Eps8, Akt, p-Akt, Erk and p-Erk protein expression in MDA-MB-468 and H460 (NSCLC cell line) cells after EE02 treatment for 24 h. The Akt, p-Akt, Erk and p-Erk protein levels were inhibited in a dose-dependent manner in these two cell lines, while the total EGFR and Eps8 protein levels were unaffected (Fig. 5b and c).

### Acute intraperitoneal toxicity study

**Fig. 6:**
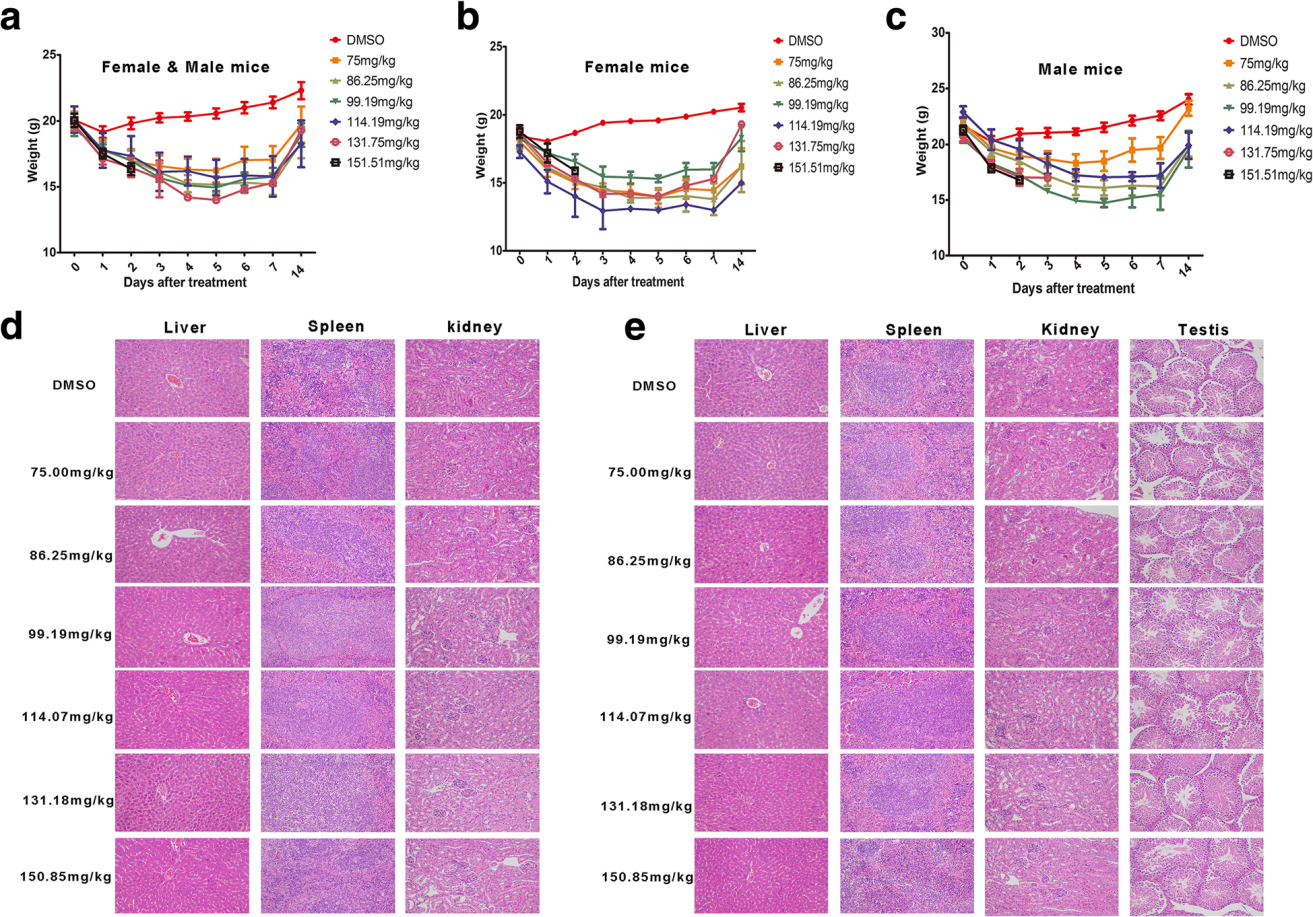
Acute intraperitoneal injection toxicity test in mice. SPF BALB/c mice were randomly assigned to 7 groups (*n* = 10, half male and half female mice). The mice were intraperitoneally injected with EE02 once at the indicated concentration (75.00, 86.25, 99.19, 114.19, 131.75 or 151.51 mg/kg b.w.) or injected with DMSO as a control, and the body weight of the mice was assessed every day for two weeks. **a** The body weights of the female and male mice are shown together. **b** The body weights of the female mice are shown. **c** The body weights of the male mice are shown. **d** Representative photomicrographs show the hematoxylin and eosin (HE)-stained liver, spleen and kidneys of the female mice intraperitoneally injected with EE02 (H&E 200×). **e** Representative photomicrographs show the HE-stained liver, spleen, kidneys and testes of the male mice intraperitoneally injected with EE02 (H&E 200×)

The results of the intraperitoneal single-dose toxicity study are summarized in Table 1. No mice died after receiving an intraperitoneal dose of up to 75.00 mg/kg b.w. EE02. Conversely, both female and male mice died when given an intraperitoneal dose of 150.85 mg/kg b.w. EE02. In other groups, different numbers of mice died, and as the dose increased, the number of mice that died gradually increased (Table 1). According to these results, the approximate LD_50_ in the mice was determined to be 101.92 mg/kg b.w. by the Bliss method, and the 95% confidence interval was 93.67 mg/kg b.w. to 110.49 mg/kg b.w.. No adverse effects or clinical signs of toxicity were observed in the surviving mice during the study except for slight weight loss during the first week after the EE02 treatment. However, in the second week, the mice gradually gained weight and approached their pretreatment weights (Fig. 6a, b and c).

**Table 1:** Intraperitoneal inject single-dose toxicity of EE02 in mice

Group	*n*	Dose (mg/kg b.w.)	Logarithmic Dose	Mortality	Mortality Rate (%)
Vehicle control group	10	5 (mL/kg b.w.)	0	0/10	0
1	10	75.00	1.875	0/10	0
2	10	86.25	1.936	3/10	30
3	10	99.19	1.996	4/10	40
4	10	114.07	2.057	7/10	70
5	10	131.18	2.118	9/10	90
6	10	150.85	2.179	10/10	100

In addition to lethality, other parameters should be considered and investigated in acute toxicity studies to identify signs of toxicity or toxic potential in specific organs. Therefore, macroscopic and histological analyses were performed in the liver, spleen and kidneys. In addition, the testes of the male mice were also examined. Macroscopic analysis showed no changes in the color or morphology of the liver, spleen, kidneys or testes when the mice were treated intraperitoneally with the different doses of the inhibitor EE02 or DMSO. Further histological analysis showed no gross pathological findings and no significant differences in the liver, spleen, kidneys or testes between the EE02- and DMSO-treated mice (Fig. 6d and e).

The acute toxicity study for EE02 was conducted in mice to establish the potential for acute toxicity and to provide information pertaining to the upper dose limit for longer-term feeding studies. On the basis of the toxicity study results, the estimated LD_50_ of EE02 in mice is 101.92 mg/kg b.w. Under the conditions of this study, a unique composition of EE02 and DMSO did not produce any acute intraperitoneal toxicological effects. This indicates that EE02 has relatively low toxicity and high potential for development as a new drug.

### Efficacy of EE02 in an animal model

**Fig. 7:**
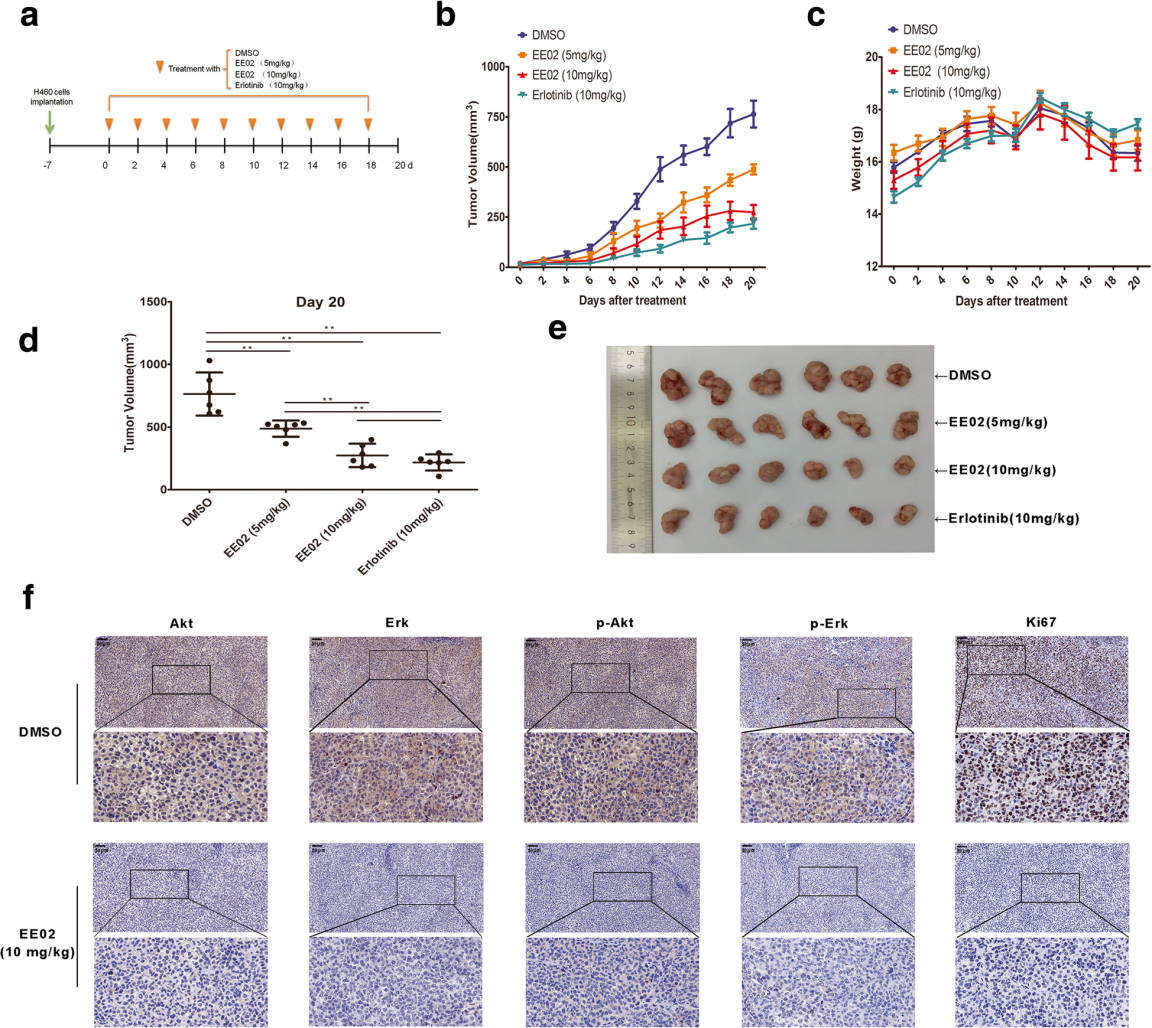
Antitumor activity of EE02 in an H460 xenograft model. **a** H460 cells (5 × 10^6^ per flank) were injected into the right flank of mice. When the volume of the tumors reached 50 mm^3^, the mice were randomly divided into four groups (6 mice in each group). Then, DMSO (negative control), low-dose EE02 (5 mg/kg b.w.), high-dose EE02 (10 mg/kg b.w.) or erlotinib (positive control, 10 mg/kg b.w.) was intraperitoneally injected into the mice every other day. **b** The volume of each tumor was measured every other day for 20 days. **c** The body weights of the mice are shown. **d** The volume of each tumor was measured after tumor extraction at the experimental endpoint. **e** The tumors excised from the mice after 20 days of treatment are shown. **f** The expression of Akt, p-Akt, Erk, p-Erk and ki67 in the tumor tissue at the end of the experiments were assessed using IHC staining

We next sought to explore whether EE02 exhibited anticancer activity in an orthotopic H460 xenograft mouse model. Compared to the DMSO treatments, the low-dose EE02 (5 mg/kg b.w., *P* = 0.003, t-test), high-dose EE02 (10 mg/kg b.w., *P* < 0.001, t-test) and erlotinib (10 mg/kg b.w., *P <* 0.001, t-test) treatments significantly suppressed tumor growth, as is shown by the photos of the tumors excised at the end of the experiment (Fig. 7e). Compared with low-dose EE02 (5 mg/kg b.w.), both high-dose EE02 (10 mg/kg b.w., *P* = 0.001, t-test) and erlotinib (10 mg/kg b.w., *P <* 0.001, t-test) significantly inhibited tumor growth in the H460 xenograft model (Fig. 7b and d). There was no difference between the high-dose EE02 (10 mg/kg b.w.) and erlotinib (10 mg/kg b.w.) treatments (*P* = 0.236, t-test) (Fig. 7b and d). We monitored mouse body weights over a 20-day period to assess toxicity and found no statistically significant differences in mouse body weight among the four experimental groups, indicating that these inhibitors have very low or no toxicity in vivo (Fig. 7c). As is shown in Fig. 7f, compared to those from the DMSO control mice, the tumors from the EE02-treated mice showed decreased proliferation, as assessed by Akt, Erk, p-Akt, p-Erk and ki-67 staining. These data demonstrated that EE02 is a potential inhibitor that is able to reduce the growth of tumors.

## Discussion

The JXM domain of EGFR is a critical region for EGFR activation and the binding site Eps8 uses to form the EGFR/Eps8 complex [13–17, 27, 43]. According to XiaolingXie et al.*,* peptide 327 derived from the Eps8 can bind to the EGFR JXM domain and effectively reduce EGFR/Eps8 complex, which thereby affects EGFR signaling and reduces cell viability [2]. Effective though, peptide inhibitors have the disadvantages of being difficulty in penetrating the membrane and the usage of high drug dosage [2, 44–46]. Quite on the contrary, using drug-like, nonpeptide small molecules to disrupt the EGFR/Eps8 complex conformation may have several advantages, including improved cell permeability, small dosage, better in vivo stability and bioavailability [30], which are more in line with clinical application requirements. In this study, we used a computational screening approach to identify potential small-molecule inhibitors of the EGFR/Eps8 complex from a database of 390,000 small molecules. Of the 29 candidate compounds tested, EE02 showed remarkable inhibition of the growth of tumor cells with high expression of EGFR and Eps8 and promoted apoptosis, while did not significantly inhibit and promote apoptosis in normal cell lines (Fig. 1, Fig. 2 and Fig. 3).

Next, we proposed that the observed antitumor effects of the inhibitor EE02 could be explained by competitive protein binding between EE02 and Eps8 towards EGFR. The JXM domain of EGFR is critical for receptor activation [13–17], and targeting this region could be a promising strategy. To further confirm whether the inhibitor EE02 directly binds to the JXM domain of EGFR and disrupts the EGFR/Eps8 complex conformation, we designed a mut-EGFR in which the JXM domain of EGFR was replaced by a 30 amino acid (GGS)_10_ linker. Such mut-EGFR is very similar to and of the same length as wt-EGFR (Fig. 4a). This substitution did not affect the structure or function of the transmembrane and kinase domains. Results showed that the inhibitory effect of EE02 on the proliferation of the MCF-7 cells overexpressing wt-EGFR was significant, but there was no significant change in the proliferation of the MCF-7 cells overexpressing mut-EGFR compared with that of the MCF-7-NC cells (Fig. 4d). Such results indicate that the inhibitor EE02 directly binds to the JXM domain of EGFR and competitively blocks the formation of the EGFR/Eps8 complex.

We further hypothesized that the EGFR/Eps8 complex inhibitor could negatively affect the survival of cancer cells by suppressing the downstream signaling of EGFR/Eps8. Inhibitors that can block downstream or upstream elements in the EGFR signaling pathway have been extensively studied in cancer research over the past two decades with the reasons that EGFR downstream signaling regulates tumor progression via proliferation, angiogenesis, metastasis and drug resistance mechanisms [2, 8, 47–50]. In our study, western blotting and protein chip assays both showed that the inhibitor EE02 could downregulate the expression of Akt, p-Akt, Erk and p-Erk (Fig. 5), which firmly support our hypothesis. Our results were compatible with those of a previous study, which demonstrated that the activation of the PI3K/Akt/mTOR and MAPK/Erk pathways could promote cancer cell proliferation.

Small-molecule EGFR inhibitors, such as erlotinib and gefitinib, compete reversibly with ATP to bind to the kinase domain of EGFR and, thus, inhibit EGFR autophosphorylation and downstream signaling [48, 51, 52]. However, the structure of the kinase domain of EGFR is very unstable and can be easily mutated, which may cause problems such as drug resistance of small molecule inhibitors targeting this region [8, 53–59], thereby limiting their clinical application. Compared with the kinase domain, the JXM domain of EGFR is relatively conserved with more stable structure and is not easy to mutate [14]. This may mean that small molecule inhibitors targeting this region are not prone to drug resistance and have a better application prospect. The vivo studies provided primary evidence that the inhibitor EE02 could selectively inhibit H460 cancer cell growth in the xenograft nude mouse model. What’s more, there showed no significant difference in the antitumor effects between erlotinib and EE02 at the same dose (Fig. 7), implying that EE02 may be a novel antitumor drug.

## Conclusions

In summary, using a combination of virtual screening and biochemical assays, we have screened small molecules and demonstrated that the inhibitor EE02, a specific EGFR/Eps8 inhibitor, can bind to the JXM domain of EGFR and directly inhibit the EGFR interaction with Esp8. The present study is the first successful attempt to identify direct disruptors of the EGFR/Eps8 complex using an in silico virtual screening approach, and this approach resulted in the identification of the inhibitor EE02. On the basis of these results, more potent EGFR/Eps8 complex inhibitors should be investigated, with the goal of developing novel therapeutic strategies that can act as complementary approaches to treat EGFR-positive and Eps8-positive malignancies.

## Abbreviations

aa: Amino acids
CoIP: Coimmunoprecipitation
EGFR: Epidermal growth factor receptor
Eps8: Epidermalgrowth factor receptor pathway substrate 8
ICD: Intracellular domain
IHC: Immunohistochemical
JXM: Juxtamembrane
NSCLC: Non-small cell lung cancers
PBS: Phosphate-buffered saline
PDB: Protein data bank
PI: Propidium iodide
PTB: Phosphotyrosine binding protein
RNAi: RNA interference
RTK: Receptor tyrosine kinase
SAM-PNT: Sterile alpha-pointed
SPF: Specific pathogen-free
